# General Practice in the Time of COVID-19: A Mixed-Methods Service Evaluation of a Primary Care COVID-19 Service

**DOI:** 10.3390/ijerph18062895

**Published:** 2021-03-12

**Authors:** James Hibberd, Jessica Carter, Michaella McCoy, Meena Rafiq, Amita Varma, Rita Sanghera, Philippa Matthews, Greta Rait

**Affiliations:** 1 Islington GP Federation, London N7 8EG, UK; james.hibberd@nhs.net (J.H.); jcarter@sgul.ac.uk (J.C.); michaella.mccoy@nhs.net (M.M.); amita.varma@nhs.net (A.V.); rita.sanghera@nhs.net (R.S.); philippa.matthews@nhs.net (P.M.); 2 Institute for Infection and Immunity, St George’s University, London SW17 0RE, UK; 3 Epidemiology of Cancer and Healthcare Outcomes (ECHO) University College, London WC1E 6BT, UK; Meena.rafiq@ucl.ac.uk; 4 RM Partners, London SW1H 0QS, UK; 5 Primary Care and Population Health Department University College, London NW3 2QG, UK

**Keywords:** COVID-19, severe acute respiratory syndrome coronavirus 2, pandemics, service evaluation, general practice

## Abstract

Primary care coronavirus disease 2019 (COVID-19) clinics were rapidly introduced across the UK to review potentially infectious patients. Evaluation of these services is needed to guide future implementation. This mixed-methods study evaluates patient demographics, clinical presentation, co-morbidities, service usage, and outcomes for the Islington COVID-19 service (London, UK) and from April to May 2020 and thematically analyses survey responses from 29 service clinicians and 41 GP referrers on their service experience. Of the 237 patients booked into the service, a significant number of referrals (*n* = 91; 38.6%) were made after the presumed infectious period of 14 days. Almost half of all adult referrals (49%) were dealt with remotely (via telephone/video consultation +/− remote oxygen saturation monitoring). The service was perceived to provide a safe way to see patients; it developed local expertise, learning, and empowerment; and it was a positive teamworking experience. These findings suggest that the management of many patients with COVID-19 symptoms is possible in routine general practice with minimal risk through the implementation of remote consultation methods and in patients who present after the post-infectious period. Additionally, the use of remote saturation monitoring and local GP COVID-19 “experts” can support practices to manage COVID-19 patients. Future primary care COVID-19 services should act as empowerment tools to assist GPs to safely manage their own patients and provide support for GPs in this process.

## 1. Introduction

**Figure 1:**
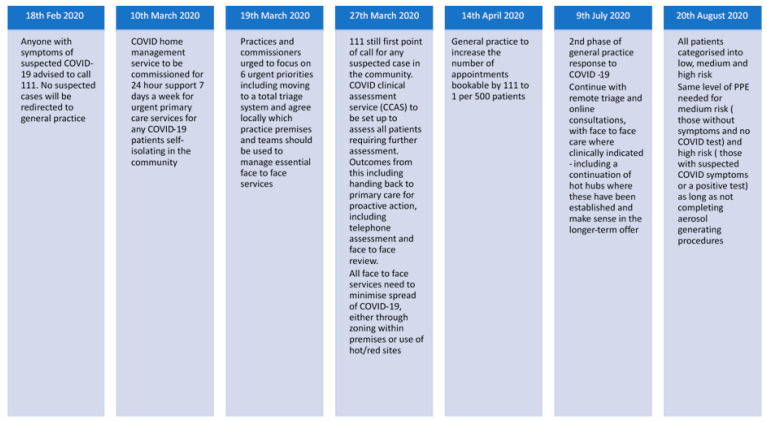
Timeline of NHS England suggested management of suspected coronavirus disease 2019 (COVID-19) within the community [5,11,12,13,14,15,16].

Severe acute respiratory syndrome coronavirus 2 has led general practice in the United Kingdom to change more rapidly in the last six months than in the last decade [1]. In the early stages of the emergence of coronavirus disease 2019 (COVID-19), there was constantly developing evidence about its epidemiology, clinical characteristics, and infectivity, which suggested that changes would be needed in usual medical practice [2,3]. Rapid digitalisation, the shift to complete telephone triage, and the creation of centralised clinical assessment services became routine following the declaration of a pandemic in March 2020 [4]. The initial response for community management of COVID-19 in the UK’s universal National Health Service (NHS) focused on utilising the national medical telephone helpline NHS 111 but quickly evolved to incorporate General Practice due to the overwhelming demand on and limited capacity of 111 (Figure 1).

General practice faced the challenge of managing suspected cases within the community while keeping infectious (“hot”) patients separate from non-infectious (“cold”) patients [5]. NHS England guidelines for achieving this were either zoning within a building or the creation of completely “hot” sites [5]. Alongside these physical changes to primary care organisation, there was also a shift, based on evidence from across the world, toward telemedicine as a way to manage potentially infectious patients [6,7,8,9]. Similar systems have been put in place in other countries with developed economies [10].

Diagnostic centers were used with varying degrees of success in Ebola outbreaks and “hot” sites were used during the H1N1 pandemic in Canada, although there is little published literature documenting their effectiveness [17,18]. During the H1N1 pandemic, primary care was found to be central to any healthcare system response [19]. This has led Australia in particular to focus on primary care during the current pandemic with GP-led respiratory clinics, although data on this approach has yet to be published [20].

Due to limited clinical evidence, there is a division of opinion about the use of community COVID-19 services. They have been described as a “20th century solution to a 21st century problem” but also as giving ‘some system resilience’ [21]. Despite the controversy, Clinical Commissioning Groups (CCGs) across the UK were tasked with the rapid roll out of primary care-led COVID-19 services [5].

We report the findings of a service evaluation of a primary care service for patients with suspected COVID-19 in Islington, London. It aimed to examine how this service was used by GPs, the type of patients seen, management, and to assess the satisfaction of both service staff and referring clinicians. Currently, there are no published studies on the use of dedicated general practice-led COVID-19 services. This service evaluation fills a crucial gap in the literature, both adding an evidence base for the future development of similar services within primary care and indicating where further research is needed [22].

## 2. Materials and Methods

### 2.1. Setting

The Islington COVID-19 service was set up to provide advice, triage, face-to-face assessment, and home-visiting. It took referrals of potentially infectious patients with suspected COVID-19 who needed an examination. The infectious period was taken to be up to 14 days after the first symptom based on best evidence, although referrals were accepted beyond this period [23].

The service evaluation was designed to capture data from the initiation of the service (18 April 2020) to its step down to a COVID-19 home-visiting service (31 May 2020).

### 2.2. Design

This is a service evaluation. Service evaluations set out to define or judge the current care within a service [24,25]. Due to the rapid development and roll out of the service, the evaluation was based on the Model for Improvement, as used by the Institute for Health Care Improvement, and the Kirkpatrick Framework for Evaluation [26,27,28]. A mixed methods approach was used, examining patient data and online surveys of both referrers and service staff.

### 2.3. Public and Patient Involvement

Due to the speed at which the evaluation was done, this service evaluation did not incorporate public and patient involvement; we recognise this as a limitation, and the findings will be disseminated at a local patient and public involvement group later in the year.

### 2.4. Patient Data

All patients referred into the service from its initiation to step down to a home visiting service were included in this evaluation. There were no exclusion criteria. A data extraction tool was designed in excel. Anonymised data were extracted by service-based clinicians from review of the GP electronic health records of referred patients. Data were collected on patient demographics (age, sex, ethnicity, smoking status) and co-morbidities, including those thought to increase risk of severe COVID-19 (diabetes, hypertension, asthma, coronary heart disease (CHD) and chronic obstructive pulmonary disease (COPD) [23].

Data were collected on patient presentation: presence or absence of key symptoms (e.g., cough, temperature, anosmia), duration of symptoms, management pathway, and outcome of assessment (i.e., if patients were triaged by phone alone, seen face-to-face on site, or visited at home) [23]. Where full outcome of assessment data was not available, these were assessed as lost to follow up.

Descriptive analyses were conducted on adults and children separately. Data were cleaned and exported from excel data extraction tool to STATA (SE 16). Data were analysed and are presented using percentages, means and standard deviations, medians and interquartile ranges (IQR). 

**Figure 2:**
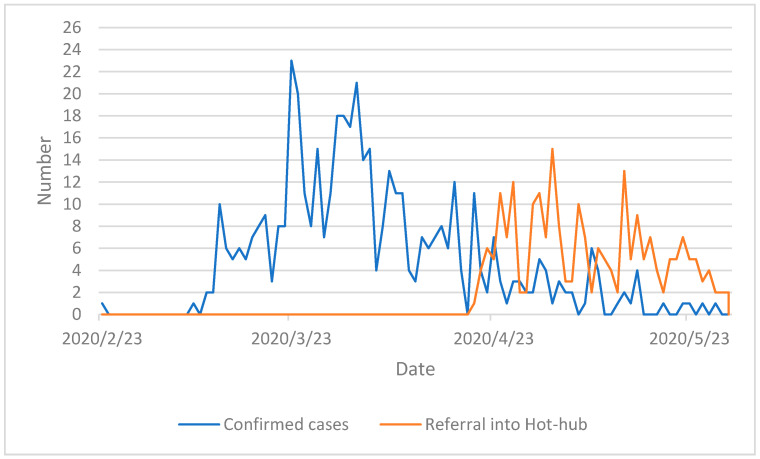
Number of new PHE lab confirmed COVID-19 cases in Islington and new referrals into the Islington Service [30].

For each patient, the date of the initial referral was recorded and used to plot the frequency of referrals over time compared to Public Health England (PHE) data on laboratory confirmed cases of COVID-19 in Islington [29] as demonstrated in Figure 2.

### 2.5. Online Surveys

Two surveys were created to gather feedback from service staff and referrers. The surveys were developed in an iterative fashion by the core evaluation team (and included a combination of tick-box responses and free text questions. The questions were refined following feedback from extended members of the team. The surveys were piloted on 4 members of staff. The final questionnaire was distributed through the web-based application survey monkey. The survey was set so there could not be more than one response from the same IP address.

The staff survey (Supplementary S1) included questions on staff demographics, reasons for working for the service, what worked well and what could be improved, and how Islington should respond to a second wave. The referrer survey (Supplementary S2) included questions on referrer demographics, reasons they referred patients in, what the service did well and how it could improve, and what was impacting on their level of confidence to see patients at their own practices. Surveys were sent to all staff and referrers and a reminder email was sent a week later.

Descriptive analysis was performed of the quantitative survey responses and the free text responses were analysed thematically each respondent being given a participant ID e.g., staff 01/ referrer 01. A coding framework was developed and emerging themes were discussed and agreed upon with the wider evaluation team.

## 3. Results

### 3.1. Patient Referral Data

In total, 201 adults and 36 children were referred to the COVID service during the evaluation period. The referral pattern into the service is mapped against confirmed COVID-19 cases in Islington in Figure 2.

### 3.2. Patient Characteristics 

Among adult patients, females were more commonly referred into the service than males (63% vs. 37%). Of those adult patients referred who had ethnicity recorded, 52% were from Black, Asian, and Minority Ethnic groups (BAME), whereas the BAME groups make up about one-third of the Islington population (32%) [31]. The percentage of patients who had ever smoked was 43.8%, which was similar to the most recent data from 2015 in Islington of 45.1% [32]. The median age of adults was 52 years old (IQR 29, range: 19–100 years) and 2 years old for children (IQR 8, range: 0–15 years). (For full demographics, see Figure S1). Recorded co-morbidities were also documented and demonstrated in Table 1.

### 3.3. Symptoms at Presentation 

Typical symptoms at presentation and frequency were recorded and can be seen in Table 2. The median period from the start of adult patient symptoms to referral was 14 days (IQR 20.5, range 1–112), and for children, it was less at 3 days (IQR 7.5, range 1–42). Over one-third of patients 38.6% (*n* = 91) were referred to the service after the presumed infectious period of 14 days from symptom onset.

### 3.4. Referral Outcomes

**Figure 3:**
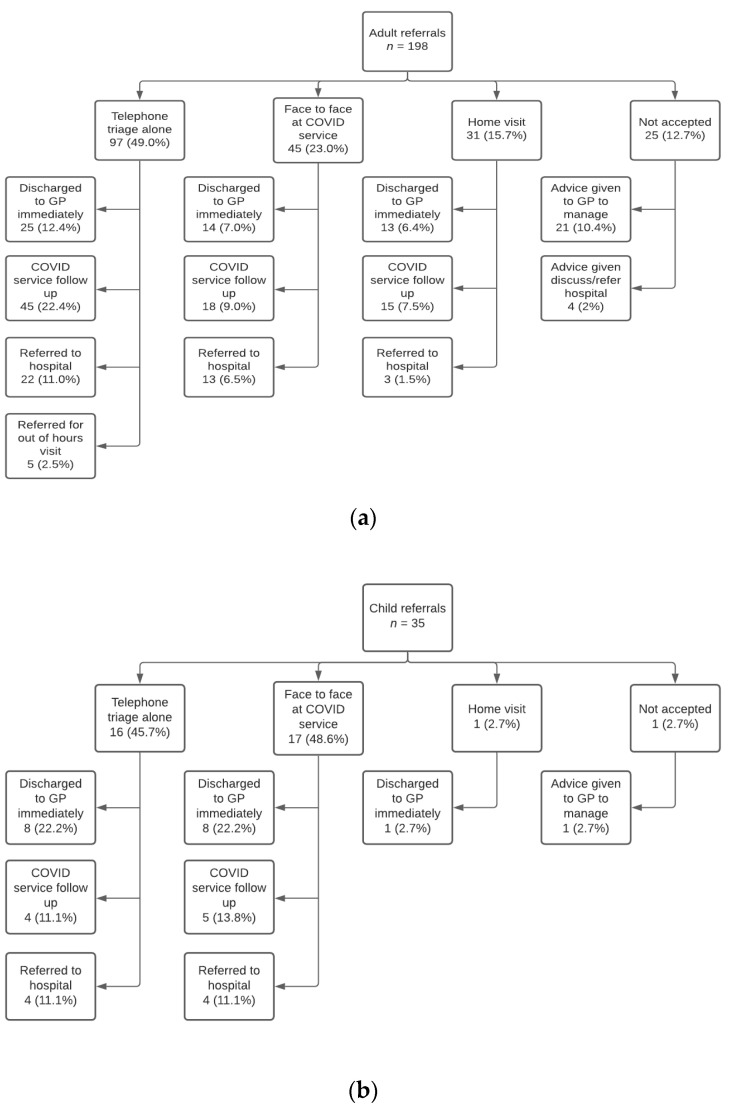
(**a**) flow diagram showing the clinical outcomes of adult cases referred into the COVID-19 service. Three adults lost to follow up, (**b**) flow diagram showing the clinical outcomes of child cases referred into the COVID-19 service. One child lost to follow up.

Approximately half of adult and child referrals were dealt with remotely via telephone and video triage alone by the COVID-19 service, 49% and 46% respectively Full referral outcomes can be seen in Figure 3a,b.

### 3.5. Online Surveys

There were 41 responses to the GP referrer survey and 29 responses to the staff survey from the 41service staff (response rate 70.7%). All (100%) survey respondents answered at least one white space question.

The top three reasons given for lack of confidence in managing COVID-19 given by referring GPs were inability to zone in current premises (54.8%); personal health concerns (29.0%); and lack of confidence in quality and use of personal protective equipment (PPE) (29.0%).

Over half (65.5%) of service staff and 87.5% of GPs who responded to the survey felt that in the event of a second wave, a specific COVID-19 service would be the best model.

#### 3.5.1. Safety of Systems at the COVID-19 Service 

Referrers and service staff expressed that the COVID-19 service offered a safer way of seeing patients than possible in surgeries, naming concerns around inability to zone in current premises and practical issues with decontamination.

It was felt that the COVID-19 service offered the ability to see “patients in a controlled environment, having adequate PPE and infection control training” (Staff03). This was contrasted with a sense that “lots of the referrals were out of fear of catching COVID, so GPs avoided any contact” (Staff03).

#### 3.5.2. Furthering Local Expertise, Learning, and Empowerment 

There was a clear agreement from both surveys that the COVID-19 service provided a way for the clinicians working there “to gain experience” (Staff 04) but also to “share local working knowledge and knowledge” (Staff 03). Links between the COVID-19 service and secondary care were seen as an educational tool allowing “sharing [of] knowledge between fellow GPs and hospital consultants” (Staff 06).

There was a feeling that this knowledge could be used to empower local GPs, “the real strength is in enabling GPs to see patients themselves” (Referrer 25); “most practices should be empowered to see their own patients” (Staff 06). It was suggested that the service was creating local experts: “I think if COVID cases are collected in one place, the doctors are more skilled/experienced” (Referrer 38).

#### 3.5.3. Teamworking

The collaborative working engendered by the COVID-19 service was seen as a positive alternative to solo GP working.

Referrers felt that it was “helpful to discuss cases with another GP” (Referrer 28) and that this allowed “shared decision making with GP colleagues” (Referrer 04) as well as being “better for doctors’ morale, and we should do more of this in our usual practice” (Staff 11).

#### 3.5.4. Missing the Peak

There was a recognition that the service had been set up after the peak of infections, “it started as the first wave had already tailed off and case numbers were low” (Referrer 40). Some had felt this had led to “low levels of activity” (Staff 02).

## 4. Discussion

### 4.1. Summary

A substantial proportion (38.6%) of patients were referred to the COVID-19 service after the presumed infectious period; many of these were seen with post COVID-19 respiratory symptoms (shortness of breath, chest pain) rather than acute infection [33]. Almost half of adult referrals (49%) were dealt with remotely with use of telephone and video assessment and delivery of saturation probes.

The benefits of the COVID-19 service reported were its ability to see potentially infectious patients in a safe environment, as well as promoting teamworking and the spread of education to empower local GPs. Both the survey and the quantitative data demonstrated that the service had been set up after the peak of infection in Islington.

### 4.2. Strengths and Limitations

To the best of our knowledge, this evaluation provides the first service evaluation of a primary care COVID-19 service. In the UK, there is an ongoing Oxford University study assessing models of care in the COVID-19 pandemic, but this will not publish for some time [22]. Detailed review of patient notes allowed large amounts of data to be collected from a relatively small patient group. There was a good response to the survey request with responders providing free text responses allowing for thematic analysis.

Rapid setup of the service led to data collection tools being created as the service was running rather than planned beforehand. This may have led to some useful data being missed. Additionally, as the referrer survey was sent widely to GPs, we do not know the true response rate, and GPs who did not refer may have been less likely to answer. As the service was set up before widespread community testing for SARS-COV2 was available, there is a lack of testing data to confirm diagnosis.

Other limitations include the lack of patient feedback. As end users, their input would have been valuable. Likewise, as we were working closely with secondary care, colleagues’ feedback from relevant consultants would also have been useful. Data were not extracted on all long-term conditions mentioned in the PHE disparities report, as this was published after data collection was completed [34]. Most significantly, we did not include chronic kidney disease (CKD) and dementia. Future research studies should include testing data, patient perspectives, and patient and public involvement, and these should be incorporated before a service is established.

### 4.3. Comparisons with the Literature

There is some evidence from Australia and Canada suggesting that Influenza Assessment Clinics (IAC) during the 2009 H1N1 outbreak reduced the burden on Emergency Departments [18,35]. These were not primary care led and, therefore, were a different model than that discussed in this paper. During the current pandemic, there have been GP-led COVID-19 respiratory clinics set up in rural Australia that are having some successes [36,37]. However, it is too early for any formal evaluation of these services to have been published.

### 4.4. Implications

The evidence around the timing of set-up suggests that any future service should have a flexible workforce allowing it to be expanded at short notice in the event of a spike in infection rate.

Our data show that a substantial proportion of referrals to the service were made after the presumed infectious period. This may reflect that GPs were being faced with the later more complicated sequelae of COVID-19 and were looking for help from a service that was perceived as more “expert”. This was not the service’s intended function, and more recently, post-COVID respiratory clinics have been set up to address this need. Many of these patients could be safely triaged in routine primary care. Any further COVID-19 services need to have a clear separation and referral pathways for acute and chronic presentations.

The ability of the COVID-19 service to manage a large proportion of referrals remotely highlights the importance of the educational and teamworking aspects of the service as well as the use of technology. The implication being that with the correct education support, access to real-time case discussions, and remote monitory technology (video consultations and saturation probe delivery), GPs could be empowered to manage the majority of their COVID-19 patients in-house.

It was clear from survey data that staff felt safe working in the COVID-19 service. Whilst concerns about the suitability of GP premises to see infectious patients are not easily remedied, concerns about personal health and lack of confidence using PPE are surmountable with risk assessment and training. Given evidence of asymptomatic transmission, it is now accepted that infection prevention and control procedures (IPC) should be the same for all patients seen in primary care, as their risk of carrying COVID-19 is much the same [16,38]. Following the lack of PPE provision to primary care at the start of the pandemic, if we are to safely see patients in individual practices moving forwards, infection control provision and training needs to be watertight [39].

Given the success of the service as an educational tool, there may be a role for a future model to be more focused on empowering GPs by offering a telephone advice line allowing a collaborative approach to patient management. Future primary care COVID-19 services should focus on enabling GPs to review their own patients, which is well recognised as the safest model of care, by providing training in remote assessment and IPC supported by telephone and video triage [40,41]. This will free up COVID-19 service capacity to review and see patients presenting with COVID-19 symptoms who need face-to-face assessment in situations when their usual GP is currently unable to manage them, either due to workforce shortages, a lack of adequately trained staff, or a lack of PPE.

**Figure 4:**
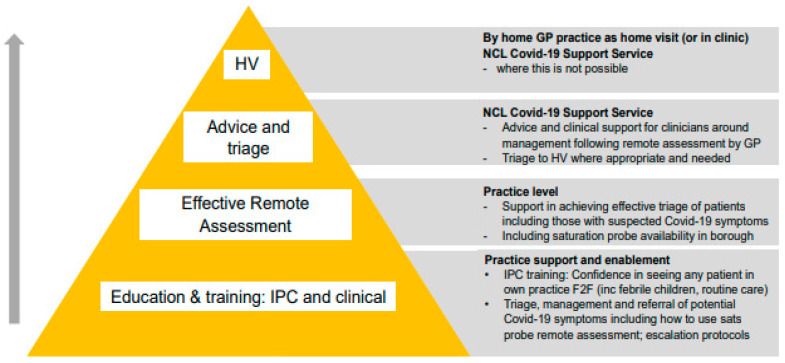
Model and principles of the North Central London (NCL) COVID-19 support service.

This evaluation has implications for clinical practice and commissioners, and data from this study has informed the service model that is now being adopted across the North Central London (NCL) CCG (Clinical Commissioning Group) (Figure 4). From an academic perspective, this service evaluation provides valuable groundwork required to develop future studies on the effectiveness of COVID-19 primary care services and any potential future pandemics; a further evaluation of the new support service is now underway.

## 5. Conclusions

Management of COVID-19 in primary care would benefit from the existence of a flexible centralised service with a focus on local GP empowerment, shared learning, facilitation of remote monitoring, and the ability to provide home visits as a response to high demand and workforce shortages.

During the current and future peaks of infection, we should use the knowledge and expertise gained including the use of digital tools, remote monitoring, and local GP COVID-19 “experts” to support practices to manage the majority of COVID-19 patients in house. This will streamline any primary care COVID-19 service as an empowerment tool for GPs to manage their patients independently and to provide support for GPs who are unable to do so.

## Figures and Tables

**Table 1:** Percentage of patients attending the COVID-19 service with recorded co-morbidities.

	Adult (*n* = 201) N (%)	Child (*n* = 36) N (%)
Diabetes	29 (14.4%)	0 (0%)
Hypertension	44 (21.9%	0 (0%)
COPD	25 (12.4%)	0 (0%)
Asthma	46 (22.9%)	1 (2.8%)
Immunosuppressive meds	5 (2.5%)	0 (0%)
IHD	8 (4.0%)	0 (0%)

**Table 2:** Frequency of symptoms on referral to the COVID-19 service.

Symptom	Adults % (*n*)	Children % (*n*)
Cough	61.5 (123)	11.4 (4)
Shortness of breath on exertion	55.7 (112)	5.6 (2)
Fever	44.8 (90)	75.0 (27)
Fatigue	35.3 (71)	22.2 (8)
Chest pain	21.9 (44)	8.3 (3)
Chest tightness	21.4 (43)	5.6 (2)
Myalgia	16.9 (34)	8.3 (3)
Loss of appetite	16.4 (33)	38.9 (14)
Headache	15.9 (32)	8.3 (3)
Diarrhoea	15.0 (30)	25.0 (9)
Sore throat	14.9 (30)	13.9 (5)
Abdominal pain	12.0 (24)	25.0 (9)
Shortness of breath on rest	10.4 (21)	0 (0)
Vomiting	5.5 (11)	5.6 (2)
Anosmia	5.0 (10)	2.8 (1)
Loss of taste	4.0 (8)	2.8 (1)
Skin rash	3.9 (8)	8.3 (3)

## Data Availability

The data presented in this study are available on request from the corresponding author. The data are not publicly available due to ethical reasons.

## Supplementary Materials

The following are available online at https://www.mdpi.com/1660-4601/18/6/2895/s1, Supplementary S1: Survey for COVID-19 Service Staff; Supplementary S2: Survey for Referrers; Figure S1: Demographic profile of patients referred to the COVID-19 service.
