# Supplementary material for: General Practice in the Time of COVID-19: A Mixed-Methods Service Evaluation of a Primary Care COVID-19 Service
Source: Int J Environ Res Public Health. 2021 Mar 12;18(6):2895. doi: 10.3390/ijerph18062895 (PMC7998968; doi:10.3390/ijerph18062895)
Supplement: Supplementary file 1 [file ijerph-18-02895-s001.pdf]

Supplementary S1: Survey for COVID-19 Service Staff

We would like to take this opportunity to thank you for working with us at the hot hub over the past two months. As this service will be evolving along with primary care's response to the COVID-19 pandemic we would be grateful for your responses to a short survey on your experiences of working at the hot hub. All responses are anonymous and should only take 5 minutes to complete. They will form part of a service evaluation which will help to inform the next steps we need to take in as part of the recovery from COVID-19 phase of primary care.

Demographics
1. Age (20-30, 31-40. 41-50, 51-60, 60-70)
2. Sex M/F/Rather not say
3. Role Partner/Salaried/Locum/GP Registrar/HCA/Reception/Nurse/Other
4. Primary Care Network (North/South/Central 1 and 2/ n/a)
5. Ethnicity (with census drop down box)

Hot Hub Experience
1. How many sessions at the hot hub did you do? (1-3/. 4-6/.7-9,/ >10)
2. Why did you decide to work at the hot hub in the first place – please choose most appropriate answers: financial reasons, ,need for shifts, increase COVID experience, duty as health care professional, PPE confidence, redeployed, other please add free text
3. What made you continue to work at the hot hub after your initial shifts? Free text
4. What about the hot hub system do you feel worked well – please give us 3 points
5. What about the hot hub could have worked better – please give us 3 points
6. What learning points have you taken away from your work here that you will apply to your work in your general practice regarding- COVID-19 and primary care – please give us 3 points
7. if the COVID hot hub was restarted in response to another wave would you work here again. Y/N then free text why
8. If there is another wave of cases would the hot hub be the best model to serve Islington primary care? (Y/N) then free text – if yes why, if no why and what other model can would you suggest
9. Any other comments you have about the service? Free text box

Optional Questions
1. Optional question – prior to working at hot hub had you had suspected or proven COVID-19- Y/N
2. Optional question – whilst working at the hot hub did you develop proven or suspected COVID-19 Y/N

Supplementary S2: Survey for Referrers

We would like to take this opportunity to thank all practices for supporting the hot hub for the last two months. As this service will be evolving along with primary care's response to the COVID-19 pandemic we would be grateful for your responses to a short survey on your experiences of referring into the hot hub. All responses are anonymous and should only take 5 minutes to complete. They will form part of a service evaluation which will help to inform the next steps we need to take as part of the recovery from COVID-19 in primary care.

Demographics
1. Age (20-30, 31-40. 41-50, 51-60, 60-70)
2. Sex M/F/Rather not say
3. Role Partner/Salaried/Locum/GP Registrar/HCA/Reception/Nurse/Other

4. Primary Care Network (North/South/Central 1 and 2/ n/a)

Referrer Experience
1. Did you do any shifts at the hot hub? Y/N
2. Did you refer any patients to Hot hub? Y/N
If Yes go to question 3,4,5,6:
If No go to question 7 and 8
3. How many patients do you think you referred: (1-5, 5-10, 10+)
4. What reason did you refer patients to the hot hub? – tick all that apply
a. Unable to access SATS probe
b. Patient/GP unable to use video consultation
c. Felt needed F2F assessment, not just oxygen saturations
d. Wanted hot hub GPs opinion on COVID related symptoms
e. Other (freetext)
5. What about the hot hub system do you feel worked well – please give us 3 points
6. What about the hot hub could have worked better – please give us 3 points
7. Why did you not refer any patients to the hot hub?
a. Geographically not suitable for patients
b. Comfortable seeing own COVID-19 patients face to face in practice
c. Not clinically needed - I have been able to manage using telephone
and video assessments with sats probes
d. Did not see any suitable patients to refer
e. Other – free text
8. Would there be a different model of service you would have used instead? If Y
please elaborate?
9. As the hot hub service comes to an end, on a scale on 1-10 how confident do you
feel in seeing patients with suspected COVID-19 in practice: 1-10
10. If you do not feel confident what are the reasons for this
a. Lack of PPE training
b. Lack of confidence in PPE
c. Unable to zone effectively in current premises
d. Personal health concerns
e. Lack of confidence in managing COVID-19
f. Other: (freetext)
11. If there is another wave of cases would the hot hub be the best model to serve
Islington primary care? (Y/N) then free text – if yes why, if no why and what other model
can would you suggest
12. Any other comments you have about the service? Free text box
Optional Questions
1. Optional question – have you had suspected/proven COVID-19- Y/N

Figure S1: Demographic profile of patients referred to the COVID-19 service

	**Adult (n=201)**	**Child (0-15y) (n=36)**
Age in years (IQR)	52 (IQR 29)	2 (IQR 8)
Sex		
Female	126 (62.3%)	20 (55.6%)
Male	91 (37.7%)	16 (44.4%)
Ethnicity		
Caucasian	65 (32.3%)	4 (11.1%)
Black, Asian and Minority Ethnic (BAME)	72 (35.8%)	3 (8.3%)
Missing data	64 (31.8%)	29 (80.5%)
Smoker		
Non-smoker	107 (53.2%)	n/a
Ex-smoker	53 (26.4%)	n/a
Current smoker	35 (17.4)	n/a
Missing data	6 (3.0%)	n/a
Shielding	9 (4.5%)	0 (0%)
